# Development of a Prognostic Scoring System for Hepatocellular Carcinoma Patients With Main Portal Vein Tumor Thrombus Undergoing Conventional Transarterial Chemoembolization: An Analysis of 173 Patients

**DOI:** 10.3389/fonc.2021.671171

**Published:** 2021-08-26

**Authors:** Jing-huan Li, Xin Yin, Wen-shuai Fan, Lan Zhang, Rong-xin Chen, Yi Chen, Li-xin Li, Ning-ling Ge, Yu-hong Gan, Yan-hong Wang, Zheng-gang Ren

**Affiliations:** ^1^Department of Hepatic Oncology, Liver Cancer Institute, Zhongshan Hospital, Fudan University, Shanghai, China; ^2^Key Laboratory of Carcinogenesis and Cancer Invasion, Ministry of Education, Shanghai, China; ^3^National Clinical Research Center for Interventional Medicine, Shanghai, China; ^4^Department of Orthopedics, Zhongshan Hospital, Fudan University, Shanghai, China; ^5^Department of Orthopedics, Ruijin Hospital, Shanghai Jiaotong University School of Medicine, Shanghai, China

**Keywords:** hepatocellular carcinoma (HCC), portal vein tumor thrombus (PVTT), transarterial chemoembolization (TACE), overall survival (OS), prognosis, model

## Abstract

**Background:**

Patients with hepatocellular carcinoma (HCC) with main portal vein tumor thrombus (mPVTT) have poor prognosis. Promising systemic therapies, such as target therapies, have limited benefits. The purpose of this study is to retrospectively evaluate the benefits of conventional TACE (c-TACE) and to establish a prognostic stratification of HCC patients with mPVTT.

**Methods:**

This is a single center retrospective study conducted over 5 years (duration of performing c-TACE), on consecutive HCC patients with mPVTT receiving c-TACE. Univariable and multivariable analysis were used to explore factors independently associated with overall survival (OS). Based on Cox-regression analysis, prognostic models were developed and internally validated by bootstrap methods. Discrimination and performance were measured by Akaike information criterion, concordance index, and likelihood ratio test.

**Results:**

A total of 173 patients were included. Median OS was 6.0 months (95%CI: 3.92~8.08). The independent variables correlated with survival were largest tumor diameter, tumor number, mPVTT extension, and AFP. In the final model, patients were assigned 2 points if largest tumor diameter ≥8 cm, or tumor number ≥2, 1point if main trunk was complete obstructed, or AFP ≥400 ng/ml. By summing up these points, patients were divided into three risk groups according to the score at the 15rd and 85th percentiles, in which median OS were 18, 7, and 3.5months, respectively (p<0.001). The model shown optimal discrimination, performance, and calibration.

**Conclusions:**

c-TACE could provide survival benefits in HCC patients with mPVTT and the proposed prognostic stratification may help to identify good candidates for the treatment, and those for whom c-TACE may be futile.

## Introduction

Hepatocellular carcinoma (HCC) is a global health problem and the fifth leading cause of cancer-related death worldwide (1). Due to improvements in surveillance procedures, diagnostic tools, and therapeutic options, diagnosis of early HCC is feasible in 30-60% of cases (2). However, a substantial part of patients still present portal vein tumor thrombus (PVTT) either at onset of the disease or as a result of HCC recurrence or progression, leading to an advanced stage of disease with an expected survival around 3 months with the best supportive care (3).

Sorafenib has been considered as the standard treatment for advanced HCC by European Association for the Study of the Liver (EASL), American Association for the Study of Liver Diseases (AASLD), and Asian Pacific Association for the Study of the Liver (APASL). However, median survival in patients with advanced HCC treated with sorafenib remains limited to 6.5 - 10.7 months, which is about 6.5 months in Asian patients (4), and the overall response rate (ORR) was about 4% (5). Also, the side effects of sorafenib usually lead to early treatment interruption. Therefore, beyond current recommendations, multiple efforts have been invested in expanding therapeutic options for selected patients (3).

Improvements in superselective TACE and perioperative management have made broader selection criteria for HCC treatment. In patients with PVTT receiving c-TACE treatment, the reported median OS ranged from 7 to 10 months, a range similar to studies that led to the approval of sorafenib (6, 7). Though superiority of TACE compared with sorafenib is not well-established, the addition of TACE has been proven safe and effective (8). And TACE has been recommended for advanced HCC by National Research Cooperative Group for Diagnosis and Treatment of Hepatocellular Carcinoma with Tumor Thrombus of China (9).

Nevertheless, c-TACE still appears to be contraindicated in HCC patients with PVTT involving the main portal trunk, due to the highly potential risk of hepatic failure following liver ischemic injury. However, a meta-analysis reported that less than 1% of these patients suffered from liver failure after TACE, and TACE response rates according to mRECIST were similar for both branch portal vein and main portal trunk (6). On the other hand, in real word practice, the patients with mPVTT usually have large tumor burden which is main obstacle to response to systemic target therapy. Therefore, as an accessible therapy with a more than three decades of clinical experience, c-TACE remains a crucial therapeutic option for patients with mPVTT. And an easy-to-use system needs to be developed to select the most suitable HCC patients with mPVTT for c-TACE.

The aim of current study is to identify the prognostic factors with relevant impact on patients’ overall survival, in a prospectively collected series of HCC patients with mPVTT treated with c-TACE, in order to build an easy-to-use prognostic stratification that may allow to select patients who would benefit from c-TACE.

## Materials And Methods

### Study Population

The study was approved by the Ethics Committee of Zhongshan Hospital, Fudan University (Approval No: B2021-245). It conformed to the ethical principles for medical research of the Declaration of Helsinkin. The informed consent was waived for the retrospective nature of the study.

We performed this retrospective analysis using a prospective database in patients diagnosed HCC at the Department of Hepatic Oncology, Liver Cancer Institute, Zhongshan Hospital, Fudan University. Patients were diagnosed according to biopsy examination or the American Association for the Study of Liver Diseases imaging criteria.

The inclusion criteria consisted of: 1) patients who received c-TACE as first-line therapy from January 1, 2009 to December 31, 2013, radical treatment, including surgical resection and radiofrequency ablation before c-TACE still included; 2) radiologic evidence of mPVTT on contrast-enhanced computed tomography (CT) or magnetic resonance (MRI) images as described classification (10); 3) patients with preserved liver function (Child-Pugh score ≤7) and performance status (score ≤1). Complete mPVTT was defined as complete obstruction of main portal vein by thrombus presenting as complete filling defect based on enhanced CT/MRI image of portal-phase.

Excluded criteria included: 1) patients with HCC extrahepatic metastases or comorbidity with other malignancies; 2) receiving subsequent loco-regional therapies, or concurrent systemic treatment with c-TACE; 3) absence of baseline imaging information. Finally, a total of 173 HCC patients with mPVTT were enrolled in this study (Flow chart was shown in **Supplementary Figure 1**).

### Treatment Procedure

c-TACE was performed as the standard modality of the institution (11). Briefly, the aimed tumor feeding artery was catheterized with a 4-5-Fr RH catheter, and microcatheters were used if necessary. Chemotherapy drugs, such as oxaliplatin (100-150mg), 5-fluorouracil (500-1000mg), and mitomycin C (10-20mg) or epirubicin (20-50mg), as well as lipiodol injection (5-20ml) were selected according to tumor size, vascularity, and basic liver function. For some patients with arterioportal shunt or prominent hypervascularity, gelatin sponge particles were selected.

All patients underwent routine follow-up after the initial c-TACE procedure as previously reported (11). Re-treatment c-TACE was scheduled on demand if there was confirmed residual tumor and without contraindication. Specific re-treatment interval and drug dose were adjusted based on clinical and laboratory findings (liver function, bone marrow function, tumor situation, *etc.*).

Major complications were reported per c-TACE procedure, which defined as those that were life-threatening within 30 days or resulted in extended hospitalization.

### Statistical Analysis

Quantitative baseline data were presented as median with interquartile and categorical data were presented as counts with percentages unless indicated otherwise.

Overall survival (OS) was the primary endpoint in this analysis, defined from the date of the initial c-TACE to the data of death from any cause. Patients who were alive at the last follow-up (May 31, 2015) or lost to follow-up were considered as censored data. Kaplan-Meier survival curves were used to estimate median OS and the survival rate and compared by log-rank test.

Continuous data were transformed in categorical data. Optimal cutoff point for continuous data was based on normal reference values, relevant cutoff as reported previously, and the results of maximally selected rank statistics from R package “maxstat”. Variables founded as significant (*p*<0.05) on univariate analysis were entered into the multivariate Cox proportional hazard regression analysis. A stepwise backward selection model was used to identify independent prognostic factors. The proportional hazard assumption was checked using Schodenfeld residuals test and plot of the final model.

Prognostic score was formulated by assigning ordinal scores to each of the selected factors according to the estimated Cox regression coefficient. Also, a nomogram model was established by R package “survival”, “rms”, and “nomogramEx”. Discrimination and performance were measured by Akaike information criterion (AIC), the concordance index (C-index). To measure whether the performance of the current model significantly better than other models, the likelihood ratio (LR) test was used. Calibration of the model for 3-, 6-, and 12-month OS was performed by comparing the predicted survival with the observed survival after bias correction. Bootstrap method was used for internal validation of the current score system. Re-sampled data sets of the same size as the original data sets were obtained by 1000 times random sampling with replacement. Difference (mean and 95% confidential interval) in survival rates were calculated using Kaplan-Meier estimation.

All statistical analyses were carried out using R version 3.6.1 with packages “survival”, “survminer”, “rms”, “nomogramEx”, “maxstat”, “Hmisc”, “lmtest”, and “boot”. A two-tailed *p*<0.05 was considered statistically significant.

## Results

### Baseline Characteristics

A total of 173 patients were included. Baseline characteristics were listed in **Table 1**. Most patients were male, had large lesion (≥8cm), compensated liver function, and HBV was the commonest etiological cause. All patients were in the advanced stage according to the BCLC classification (stage C). Complete obstruction of main portal vein by thrombus was found in 89 cases (51.4%).

**Table 1:** Baseline demographics and clinical characteristics for 173 HCC patients with mPVTT treated with c-TACE.

Characteristics	Number (%)/Median (IQR)
Gender	
Male	153 (88.4)
Female	20 (11.6)
Age (yr)	51 (43~60)
Aetiology	
HBV	164 (94.8)
Others	9 (5.2)
Largest tumor diameter, cm	9.5 (6.0~11.8)
<8	48 (27.7)
≥8	125 (72.3)
Tumor number	
1	88 (50.9)
≥2	85 (49.1)
Complete mPVTT	
Without	84 (48.6)
With	89 (51.4)
AFP, ng/ml	3600 (60.5~42300)
<400	60 (35.3)
≥400	112 (64.7)
Child-Pugh score	
A5	93 (53.8)
A6	63 (36.4)
B7	17 (9.8)
ALBI grade	
1	124 (71.7)
2	31 (17.9)
3	18 (10.4)
ALT, U/L	45 (30.5~67)
ALB, g/L	36 (32~39)
TBIL, umol/L	14.6 (10.1~20.9)
GGT, U/L	212 (146~326)
PT, s	13.2 (12.3~14)
c-TACE sessions	2 (1~3)

Median with interquartile range are shown for quantitative variables, whereas counts with proportions are shown for categorical variables.

mPVTT, main portal vein thrombus; AFP, alpha-fetoprotein; ALBI, albumin-bilirubin grade; ALT, alanine transaminase; ALB, albumin; TBIL, total bilirubin; GGT, gamma-glutamyl transferase; PT, prothrombin time.

### Overall Survival

The median follow-up time was 8 months. And 105 (60.7%) patients died during the time by the last follow-up day (May 31, 2015). The median survival of the entire 173 patients was 6.0 ( ± 1.06, 95% CI 3.92~8.08) months, with 6-, 12-, 24-months survival being 50%, 31%, and 16%, respectively (**Supplementary Figure 2**).

### Safety

Patients were discharged a median 4 days after c-TACE procedure (range from 2 to 25 days). Post-TACE syndrome was the most common treatment-related adverse event, and was the main reason for prolonged hospital stay. Grade 1-2 liver dysfunction were also common after treatment. But in most cases, these were reversible. There were 11 TACE-related major complications out of 373 procedures, 7 in form of grade 3-4 liver dysfunction. Other than deterioration in liver function, 1 patient with known chronic heart disease developed acute heart failure and 1 patient suffered lacunar cerebral infarction after c-TACE treatment. 2 patients died of acute tumor lysis and hepatic failure within the 30-d period following the procedure, resulting in a procedure-related mortality rate of 1.16%.

### Univariate and Multivariate Analysis

The results of the univariate analysis on patients’ baseline characteristics were listed in **Table 2**. Those seven variables identified as significant at univariate analysis were entered into a Cox-regression analysis. After stepwise removal of the variables which were not significant, maximum tumor size, tumor number, extension of mPVTT, and AFP remained as significant predictors of OS that will be considered for further model development (**Supplementary Figure 3**). The proportional hazard assumption was checked using Schodenfeld residuals test and plot of the final model (**Supplementary Figure 4**, *p*>0.05).

**Table 2:** Univariate and multivariate analysis of potential prognostic factors of OS for HCC patients with mPVTT treated with c-TACE.

Characteristics	Univariate P	Multivariate P	HR (95%CI)
Gender (female/male)	0.985		
Age >65 (no/yes)	0.881		
Largest tumor diameter (<8/≥8)	0.001	0.001	2.576(1.605~4.134)
Tumor number (single/multiple)	0.001	0.001	2.767(1.829~4.186)
Complete mPVTT (without/with)	0.002	0.005	1.800(1.192~2.719)
AFP ≥400ng/ml (no/yes)	0.005	0.024	1.626(1.066~2.480)
Child-Pugh score (A5/A6/B7)	0.566		
ALBI grade (1/2/3)	0.016		
ALT >40U/l (no/yes)	0.010		
ALB ≤3.6g/dl (no/yes)	0.309		
TB >17.1 umol/L (no/yes)	0.009		
GGT >225U/L (no/yes)	0.001		
PT >14s (no/yes)	0.507		

(n=173).

### Development of the Prognostic Model

Based on the results of the Cox-regression analysis, 2 models were developed.

**Figure 1:**
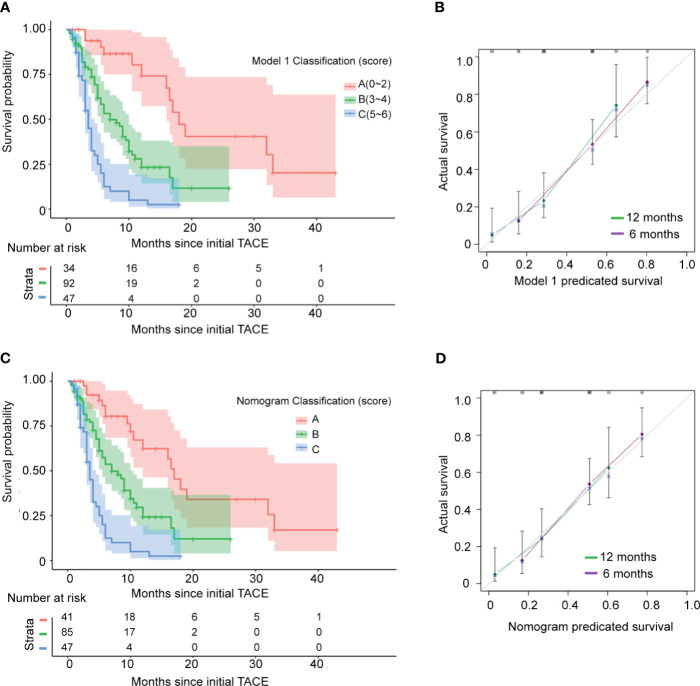
Kaplan–Meier estimated overall survival curves and calibration curves of the current staging systems in the test 173 HCC patients with mPVTT treated with c-TACE. **(A)** Kaplan–Meier estimated curves of overall survival of 173 studied patients stratified by current model 1 staging system. **(B)** Calibration curves of the model 1. The y-axis represents the actual survival rate. The x-axis represents the predicted possibility. The diagonal dashed line indicates the ideal prediction by a perfect model. **(C)** Kaplan–Meier estimated survival curves by model 2 (nomogram). **(D)** Calibration curves of the model 2 (nomogram).

To facilitate Model 1 score calculation, the estimated regression coefficients were multiplied by a factor of 2 and rounded to the nearest unit (**Table 3**). The score for Model 1 system ranged from 0 to 6. In current study group, the score identified 7 subgroups with different prognoses (**Supplementary Figure 5**, *p*<0.001). Then patients were divided into three groups according to the score at the 15rd and 85th percentiles of the scores. Group A (favorable prognosis) coincided with score 0~2, group B (intermediate prognosis) with score 3~4, and group C (dismal prognosis) with score 5~6 (**Supplementary Table 1**). Observed median OS in the three categories was 18 ( ± 1.54, 95%CI: 14.9~21.0) months, 7 ( ± 1.18, 95%CI: 4.6~9.3) months, and 3.5 ( ± 0.33, 95%CI: 2.9~4.1) months respectively (**Figure 1A**, *p*<0.001). Also, as shown in **Figure 1B**, the calibration plots of survival probability at 6- and 12-month demonstrated optimal agreement between expectation and observation.

**Table 3:** Points allocated to each prognostic factor to calculate an overall prognostic score for OS.

Prognostic factor	Model 1 points^a^	Model 2 points^b^
Largest tumor diameter		
< 8	0	100
≥ 8	2	0
Tumor number		
single	0	73.5
multiple	2	0
Complete mPVTT		
without	0	51.9
with	1	0
AFP		
<400ng/ml	0	32.7
≥400ng/ml	1	0

a Model 1 points= estimated cox regression coefficient ×2, rounded.

b Model 2 points= scores calculated form the nomogram scoring system from R package “nomogramEx”.

**Figure 2:**
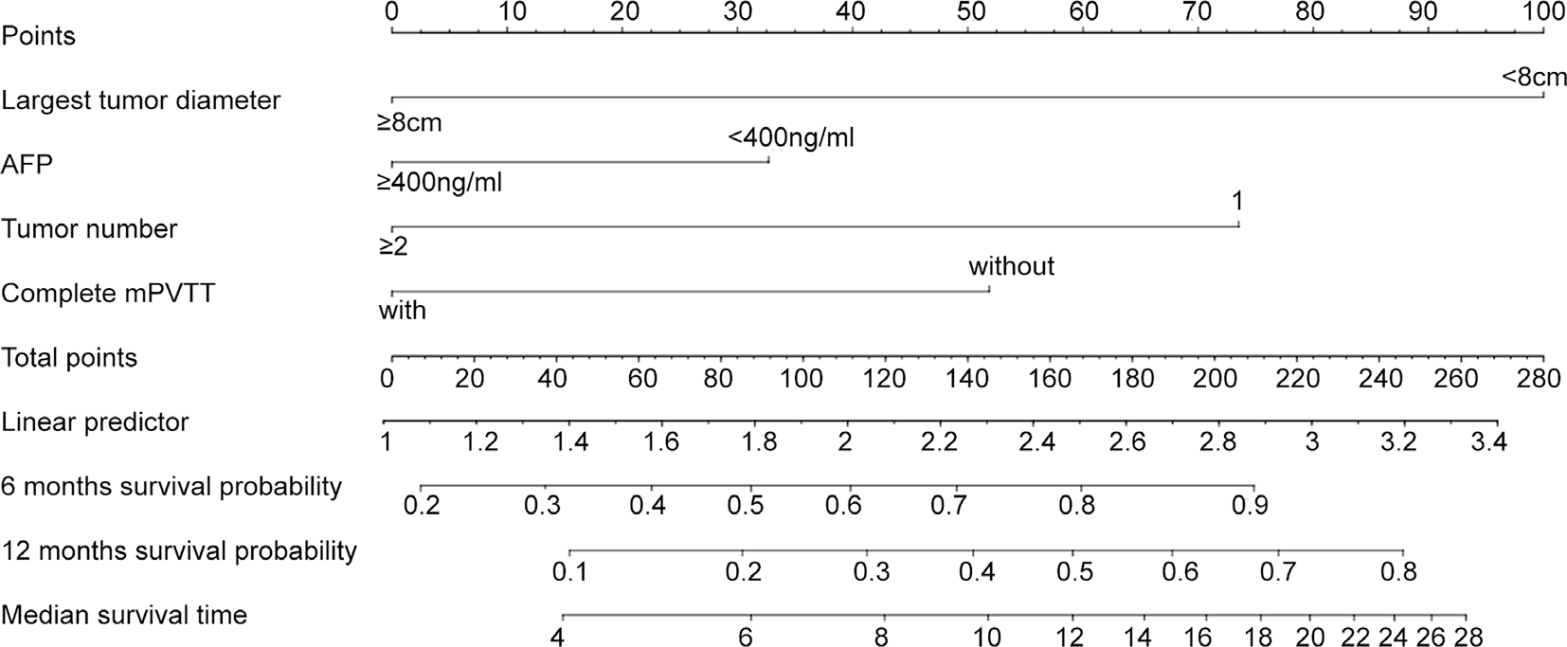
Nomograms for HCC patients with mPVTT received the c-TACE treatment. The nomogram may have the potential to individually predict survival in a particular patient according to his clinicopathologic feature and imaging signature. To use the nomogram, locate the margin according to the patient information, draw a line straight up to the points axis to obtain the score associated with each subtype within these four variables separately. The final score was obtained by adding up the total score. Locate it on the total points axis and draw a line straight down to the bottom axis, the estimated survival probability could be determined.

Based on the significant prognostic factors identified in the Cox analysis, Model 2 (a nomogram) was developed (**Figure 2**). Each subtype within these four variables was assigned a score on the point scale. By adding up the total score and locating in on the total point scale, the probability of median OS or 6- and 12-month survival rate can be estimated. As shown in **Table 3**, the nomogram scoring system can also be used for a more precise estimation of OS prediction. Optimal cutoff values for nomogram system was based on the results of maximally selected rank statistics from R package “maxstat” (**Supplementary Table 1**). Patients were stratified into three subgroups with median OS of 17 ( ± 1.27, 95%CI: 14.4~19.5), 7 ( ± 1.22, 95%CI: 4.6~9.4), and 3.5 ( ± 0.33, 95%CI: 2.9~4.1), respectively (**Figure 1C**, *p*<0.001). The calibration plots of survival probability at 6- and 12-month proved that the predictions were in good agreement with the actual observations (**Figure 1D**).

AIC, C-index, and LR test of the two possible models were listed in **Table 4**. And considering the purpose of developing an easy-to-use bedside classification tool, Model 1 was selected as the final model.

**Table 4:** Comparison of the performance and discrimination ability of current models and other systems.

Model/system	AIC	C-index	LRT loglik
Model 1	850.54	0.70	-423.53^REF^
Model 2 (nomogram)	855.23	0.70	-425.62**
HAP	887.04	0.62	-441.52***
mHAPII	884.04	0.57	-440.36***
Six-and-twelve	887.99	0.60	-443.39***
ITA.LI.CA system	893.84	0.57	-445.92***
ALBI	887.49	0.61	-441.99***

ACI, Akaike information criterion; LRT, likelihood ratio test; HAP, hepatoma arterial-embolization prognostic; mHAP, modified HAP;ITA.LI.CA system, Italian liver cancer system; ALBI, albumin-bilirubin. **p < 0.01, ***p < 0.001.

### Assessment and Comparison of the Discrimination and Performance of the Current Model and Other Models and Prognostic Systems

Discrimination was measured by AIC and C-index. The current Model 1 shown lower AIC value and higher C-index than other systems, indicating that Model 1 had higher discriminatory ability (**Table 4**). By using a LR test to compare different systems, the prognostic performance of Model 1 was consistently significantly better than the other systems (**Table 4**, *p <*0.01).

### Internal Validation

Based on the Model 1, prognosis was well distributed among the three groups (Group A, B and C) in the study population. One-year survival rates for the three groups were 63%, 25%, and 7%, respectively. Then bootstrap method was used to calculate the half- and one-year survival rate, along with 95%CI. Internal validation was performed by pairwise comparisons of the survival rates among Group A, B and C of the Model 1. As shown in **Table 5**, the lower confidence limit for difference between each pair of the Group A, B and C was greater than zero, suggesting all differences were statistically significant. Therefore, Model 1 has shown robust in estimating prognosis in distinct groups.

**Table 5:** Pairwise comparisons of the 6- and 12-month survival rates with 95%CI between each strata of the current Model 1.

Strata	6-month survival (%)		12-month survival (%)	
	Difference	95%CI	Difference	95%CI
Model1 A&B	0.201	0.151~0.285	0.410	0.331~0.463
Model1 B&C	0.395	0.301~0.437	0.173	0.009~0.213

Based on the Model 1, prognosis was distributed into three groups (Group A, B and C) among the study population. Bootstrap method was used to calculate the half- and one-year survival rate, along with 95%CI among Group A, B and C of the Model 1. Internal validation was performed by pairwise comparisons of the survival rates among these three groups. The lower confidence limit for difference between each pair of the Group A, B and C was greater than zero, suggesting all differences were statistically significant.

## Discussion

We conducted a retrospective study regarding the outcome of c-TACE for HCC patients with mPVTT. The median OS was 6.0 ( ± 1.06, 95% CI 3.92~8.08) months in current 173 patients. Significant prognostic factors for OS were maximum tumor diameter, tumor number, extension of mPVTT, and AFP.

Many oriental clinicians consider TACE as a possible treatment for patients with unrespectable HCC and PVTT, though in current guidelines the presence of any type of PVTT is considered to be the contraindication for TACE (6). Our previously published meta-analysis involving 8 comparative studies, 3 prospective and 5 retrospective studies, found TACE significantly improved the 6- and 12-month OS of PVTT patients compared with conservative treatment (12). Moreover, subgroup analysis suggested TACE was effective in patients with either segmental or main trunk PVTT (12). Although the superiority of TACE over best supportive care is confirmed by several studies, the survival greatly varied in these studies, ranging from 5 to 9 months (12). This suggests even when performance status and liver function are preserved, this population includes patients with different prognosis. And classification of these patients in clinical practice is of importance.

Sorafenib has been recognized as standard treatment in advanced HCC patients, including those complicated with PVTT. Although there are no dedicated clinical trials to evaluate the efficacy of sorafenib to PVTT patients, in the subgroup analyses of the Asian Pacific Study, the median OS of HCC patients with macrovascular invasion was about 6.5 months (4). In the setting of mPVTT, a study reported a limited median OS of HCC patient with Vp3/4 PVTT treated with sorafenib as mono-therapy was only 3.1 months (13). Besides, a study compared the efficacy of TACE and sorafenib in advanced HCC, in which 35% of patients treated with TACE with PVTT, shown no significant difference was found between these two therapies in terms of OS (14). Another multi-kinase inhibitor, lenvatinib demonstrated non-inferior surcical to sorafenib (15). However, patients with Vp4 PVTT were excluded in this study. Related to this issue are the results of our study that reported a comparable survival benefit of a consecutive series of patients with mPVTT treated with c-TACE. However, no solid data are currently comparing c-TACE with sorafenib in patients with PVTT. And it remains unclear to which kind of patients with mPVTT would truly benefit from c-TACE.

The main aim of our study was to identify the independent prognostic variables for OS in HCC patient with mPVTT treated with c-TACE and to assess whether different classifications could correlate with predictable patient outcomes.

In the study group, tumor size and tumor number were identified as important prognostic factors with higher HRs. This may because heavy tumor burden is most likely related with reduced liver function and therefore a higher risk of early liver decompensation. In addition, this situation may prevent an effective treatment by means of a single session of c-TACE, and liver dysfunction may precede re-treatments. Though tumor size has been reported as a fundamental prognostic factor of HCC patients among most candidate predictors regardless of loco-regional of systemic therapy (16–18), the current findings may be supported that better local control of primary tumor could contribute to c-TACE efficacy, which could be translated into OS benefits in patients even with mPVTT.

The effects of PVTT extent on post-treatment survival of HCC patients has been demonstrated in several literatures and our previous study (19, 20). Thrombosis in the proximal branch increases the risk of tumor spread and induces higher portal venous pressure causing elevated risk of liver failure. But most studies providing the extension of PVTT only distinguish between “main trunk PVTT” and “no main trunk involved” (6). Besides, several studies excluded patients with mPVTT (2, 15, 21). In our study, however, the results demonstrated that even in mPVTT patients, distinguish of PVTT extension (with or without completely obstructed) may still affect treatment effects thus benefit patients’ outcome.

Elevated AFP levels were shown to be associated with poor OS in patients with mPVTT in this study. It has known that a cutoff of 20ng/ml AFP have a high sensitivity in predicting HCC and 400ng/ml is a diagnostic and prognostic indicator for HCC (22). In our study, there were fewer patients with AFP less than 20ng/ml (18.1%). Thus 400ng/ml was chosen as the binary classification cutoff value.

In previous studies focusing on TACE efficacy for HCC patients, TB, GGT and ALB were reported as important factors affecting prognosis (18, 23). Unlike these studies, in our study these liver function parameters were not recognized as independent prognostic factors. It’s probably because in current practice these c-TACE treated mPVTT patients were selected to have relatively well preserved liver function. Therefore, factors related to tumor burden, tumor size, tumor number, mPVTT extension, and AFP, which were varied in a relatively large range, shown major roles in Cox regression analysis.

The feature of current study is the development of a scoring model derived from the estimated regression coefficients of the final Cox model. Based on changes in risk estimates, three classifications have been identified, allowing significant prognostic stratification in terms of OS. In particular, patients with total points less than 2 shown a relative favorable prognosis after c-TACE, with 18 ( ± 1.54, 95%CI: 14.9~21.0) months median OS, which was comparable to HCC patients receiving aggressive treatments in absence of branch or main portal trunk PVTT (6, 8, 24). When mPVTT patients scored between 3 and 4, the median OS was 7 ( ± 1.18, 95%CI: 4.6~9.3) months. Due to the comparable survival benefit of c-TACE to reported systematic treatments (4), c-TACE may be considered as an alternative treatment at this time. However, patients scored more than 5 shown a much poorer median OS than that expected with systemic treatments. c-TACE for this patients may be harmful and should be avoided.

A nomogram model was also built based on the final Cox model. From the nomogram plot, the estimated survival of the patient can be quickly understood. Meanwhile, to facilitate classification, each subtype within the four independent factors was assigned a score according to nomogram model. Similar to Model 1 we built, patients were classified into three prognostic stratifications in terms of OS. Favorable discrimination and performance were also found in the nomogram scoring model. But its performance was not better than Model 1 according to AIC and LRT loglik. Therefore, considering the simplicity of the score calculation, Model 1, which derived from the estimated regression coefficients, was finally selected.

Our study has some limitations. The retrospective nature and the relatively small size of the study limits the strength of the evidence, though it is one of the large cohorts of consecutive patients with mPVTT treated with c-TACE. Secondly, most patients had a background of HBV infection. Besides, owing to the absence of comparators, for example sorafenib therapy, the impact of prognostic stratification we discussed on treatment allocation was limited. Finally, this study lacks external validation. We hope to improve this part of work in the follow-up research.

In conclusion, our study suggests c-TACE remains a therapeutic option for HCC patients with mPVTT. Largest tumor diameter, tumor number, mPVTT extension, and AFP values have independent impacts on patients’ OS. By applying our current proposed scoring system, we have disclosed an easy-to-use model to identify HCC patients with mPVTT who may benefit from c-TACE treatment. Also, this finding has clinical implications to help avoid unnecessary c-TACE procedure associated with poor overall survival.

## Data Availability Statement

The raw data supporting the conclusions of this article will be made available by the authors, without undue reservation.

## Ethics Statement 

The studies involving human participants were reviewed and approved by The Ethics Committee of Zhongshan Hospital, Fudan University. Written informed consent for participation was not required for this study in accordance with the national legislation and the institutional requirements.

## Author Contributions 

All authors contributed to the acquisition of clinical data. J-hL and W-sF contributed to the statistical analysis. J-hL wrote the first draft of the manuscript. Z-gR supervised and oversaw the study. All authors contributed to the article and approved the submitted version.

## Funding

This study was supported by the National Natural Science Foundation of China 81802320 and 82101654, the Exploratory Clinical Research Projects of National Clinical Research Center for Interventional Medicine 2021-001 and the Foundation of Zhongshan Hospital, Fudan University 2021ZSYQ05.

## Conflict of Interest

The authors declare that the research was conducted in the absence of any commercial or financial relationships that could be construed as a potential conflict of interest.

## Publisher’s Note

All claims expressed in this article are solely those of the authors and do not necessarily represent those of their affiliated organizations, or those of the publisher, the editors and the reviewers. Any product that may be evaluated in this article, or claim that may be made by its manufacturer, is not guaranteed or endorsed by the publisher.
