# Supplementary material for: Development of a Prognostic Scoring System for Hepatocellular Carcinoma Patients With Main Portal Vein Tumor Thrombus Undergoing Conventional Transarterial Chemoembolization: An Analysis of 173 Patients
Source: Front Oncol. 2021 Aug 26;11:671171. doi: 10.3389/fonc.2021.671171 (PMC8427599; doi:10.3389/fonc.2021.671171)

**STable 1. Classification of prognostic score.**

| Model | Score |
| --- | --- |
| Model 1 |  |
| A | 0~2 |
| B | 3~4 |
| C | 5~6 |
| Model 2 |  |
| A | >151.9 |
| B | 51.0<, ≤151.9 |
| C | ≤51.9 |

Optimal cut-point for nomogram system was based on the results of maximally selected rank statistics from R package “maxstat”.

**sFIG1. Flow chart of patient selection.**


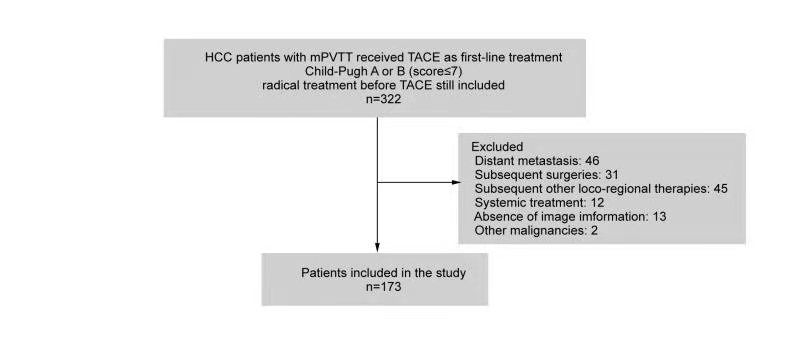


**sFIG2. Kaplan-Meier curves of overall survival of all 173 patients.**


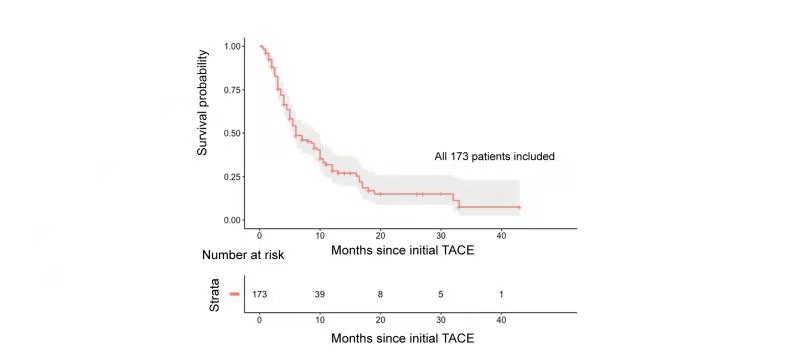


**sFIG3. Kaplan-Meier curves illustrating overall survival (OS).**

Kaplan-Meier survival curves for comparison of OS between patients with (A) tumor diameter ≥8cm and <8cm; (B) complete mPVTT and partial mPVTT; (C) multiple tumor number and single tumor; (D) AFP ≥400ng/ml and AFP <400ng/ml.


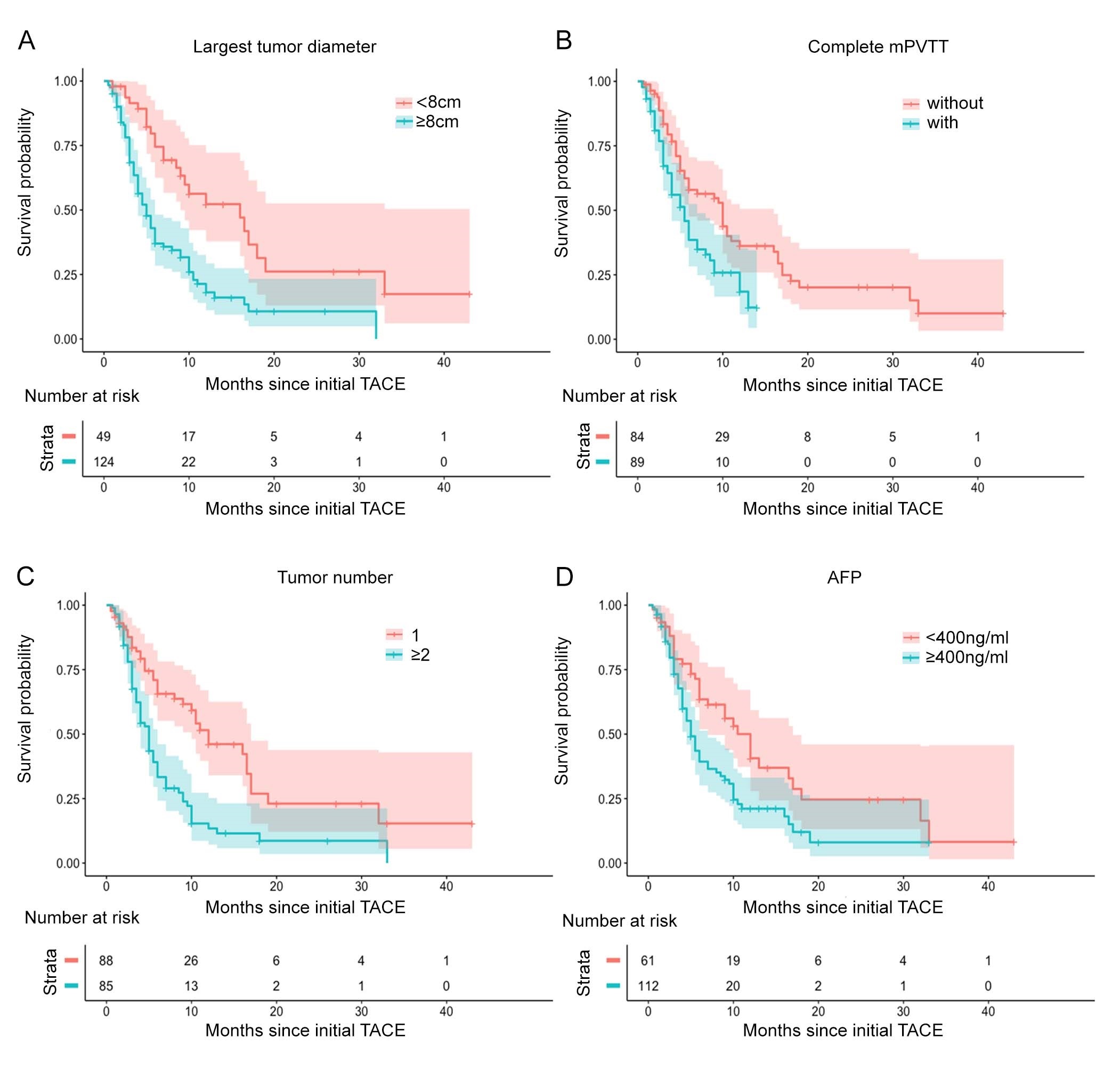


**sFIG4. The plot of Schodenfeld residuals test.**


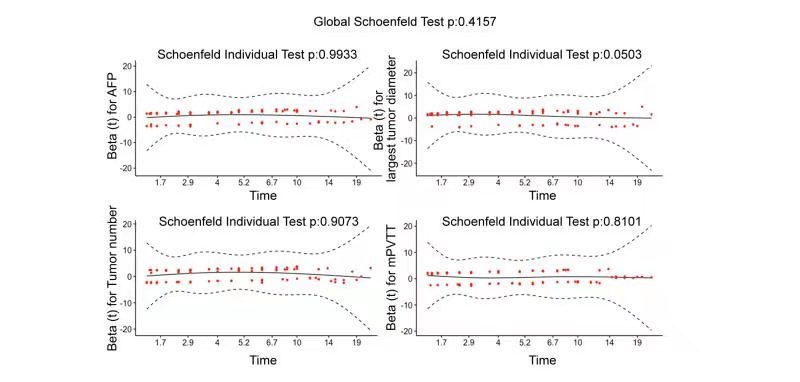


**sFIG5. Kaplan-Meier estimated curves of overall survival of 173 studied patients stratified by current model 1 score.**


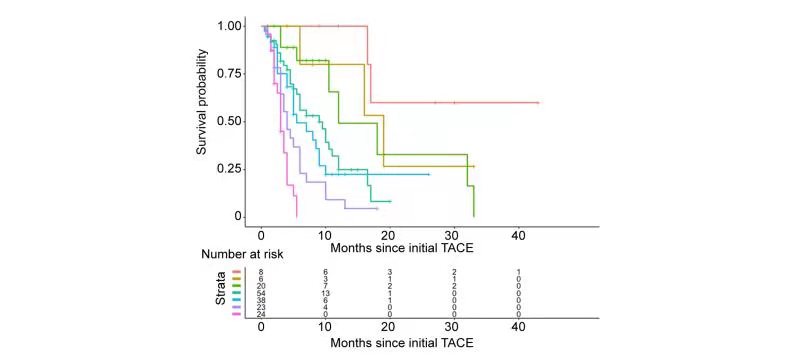

Supplement: Supplementary file 1 [file DataSheet_1.docx]
